# Effective Isolation of Picrocrocin and Crocins from Saffron: From HPTLC to Working Standard Obtaining

**DOI:** 10.3390/molecules27134286

**Published:** 2022-07-03

**Authors:** Laurynas Jarukas, Konradas Vitkevicius, Olha Mykhailenko, Ivan Bezruk, Victoriya Georgiyants, Liudas Ivanauskas

**Affiliations:** 1 Department of Analytical and Toxicological Chemistry, Lithuanian University of Health Sciences, A. Mickeviciaus Str. 9, LT-44307 Kaunas, Lithuania; laurynas.jarukas@lsmuni.lt (L.J.); konradas.vitkevicius@lsmuni.lt (K.V.); 2 Department of Pharmaceutical Chemistry, National University of Pharmacy, Valentynivska, Str. 4, 461168 Kharkiv, Ukraine; vania.bezruk@gmail.com (I.B.); vgeor@nuph.edu.ua (V.G.)

**Keywords:** saffron, picrocrocin, crocins, preparative chromatography, quality control, UPLC-ESI-MS/MS, HPTLC

## Abstract

Saffron is widely cultivated and used as a spice. Recently published data on the chemical composition and pharmacological potential of saffron determine its use in pharmacy and medicine. The proposed high-performance thin-layer chromatography (HPTLC) method allows good separation of 11 analytes. The saffron quality (Iran, Ukraine, Spain, Morocco samples) assessment was based on the European Pharmacopoeia monograph and ISO 3632. The HPTLC method for the safranal, crocin, and picrocrocin quantification was proposed and validated. The crocins content in Ukrainian saffron was from 17.80% to 33.25%. Based on qualitative and quantitative assessment results, the saffron sample from Zaporizhzhia (Ukraine) had the highest compounds content and was chosen to obtain the working standards of picrocrocin and crocins (*trans*-4GG, *trans*-2G, *trans*-3Gg) by preparative chromatography. The compounds were isolated from lyophilized extract of saffron using a Symmetry Prep C_18_ column (300 × 19 mm × 7 µm), and identified by spectroscopic techniques (HPLC-DAD, UPLC-ESI-MS/MS). The purity of crocins and picrocrocin was more than 97%. A novel method proposed to obtain working standards is simple and reproducible for the routine analysis of saffron quality control.

## 1. Introduction

The World Health Organization “Good Manufacturing Practice” (GMP) guidelines are rules that have been developed in accordance with modern pharmaceutical quality standards that establish requirements for the organization of production and the quality control of medicines.

It specifically relates to the principles of quality assurance of pharmaceuticals and specifications of medicinal substances and dosage forms [1]. These standards regulate the use of the reference standards and/or laboratory working standards as pharmaceutical reference materials [2]. Reference Pharmacopoeia Standards are chemical compounds or drug substances with high purity. They are used to perform umpire analyses and for the calibration of working standards [1,2,3]. These standards are expensive and purchased from specially certified drug laboratories, United States Pharmacopeia companies, European Pharmacopoeia, Japanese Pharmacopoeia, Swiss Pharmacopoeia, French Pharmacopoeia, Mexican Pharmacopoeia, National Institute for Standards and Testing (NIST), etc. [3,4]. Working standards can be used instead of the official reference pharmacopoeia standards for routine analyses and are an integral part of any pharmaceutical laboratory [5]. They are prepared in the workplace from certified raw materials with good repeated tests and after analysis of impurities. Working standards are used by this laboratory for routine quality pharmaceutical analysis later. Each laboratory follows the specific standards operating procedures (SOP) of working standards accepted by them to ensure the identification of the working standard. The SOP describes the procedure for the preparation and handling of the working standard [1].

Saffron is a spice derived from the red stigma of *Crocus sativus* L. (Iridaceae) and is one of the oldest natural food supplements [6]. The glycosylated apocarotenoids of crocins and monoterpene glycoside picrocrocin are accumulated in plant stigmas. The international standard ISO 3632-1:2011 is used to assess the saffron spice quality in the food industry [7]. The studies of Ukrainian saffron have shown that it meets the I quality category according to crocin, picrocrocin, and safranal content [8]. It should be noted that, in consequence of its active ingredients, saffron is used not only as a food raw material or a spice in cooking, but also as a medicinal raw material [6,9].

*Crocus* stigma has a wide variety of components, including phenolic compounds [6,10], terpenoids, apocarotenoids [7,9,11,12], amino acids [13,14], carboxylic acids [15], and others. However, the main active compounds are crocins and picrocrocin [16,17,18]. Currently, picrocrocin has been detected only in *C. sativus* stigmas [19] and it is the best authenticity biomarker in saffron [17]. Picrocrocin (C_16_H_26_O_7_) is a precursor of triterpene aldehyde safranal (C_10_H_14_O) [7,18]. Crocins (C_44_H_64_O_24_) are the water-soluble group of carotenoids of the crocetin ester family [20], including about 12 different crocin derivatives [10].

Saffron monographs are included into different countries’ pharmacopoeias [21,22,23,24,25,26,27,28] and its quality is also determined by the presence of crocin. The identification test of saffron is presented in more than 10 pharmacopoeias. However, only six of them require an analysis of the main saffron component (crocin) by thin-layer chromatography (TLC).

Recent studies showed that saffron’s main constituents (*trans*-crocin-4 and picrocrocin) have potential activity in different nervous diseases (Alzheimer’s, epilepsy, Parkinson’s, brain ischemia, memory impairment) [9,20,29], as an active anticancer agent [16], and also as a potential anti-aging effect [30].

**Figure 1:**
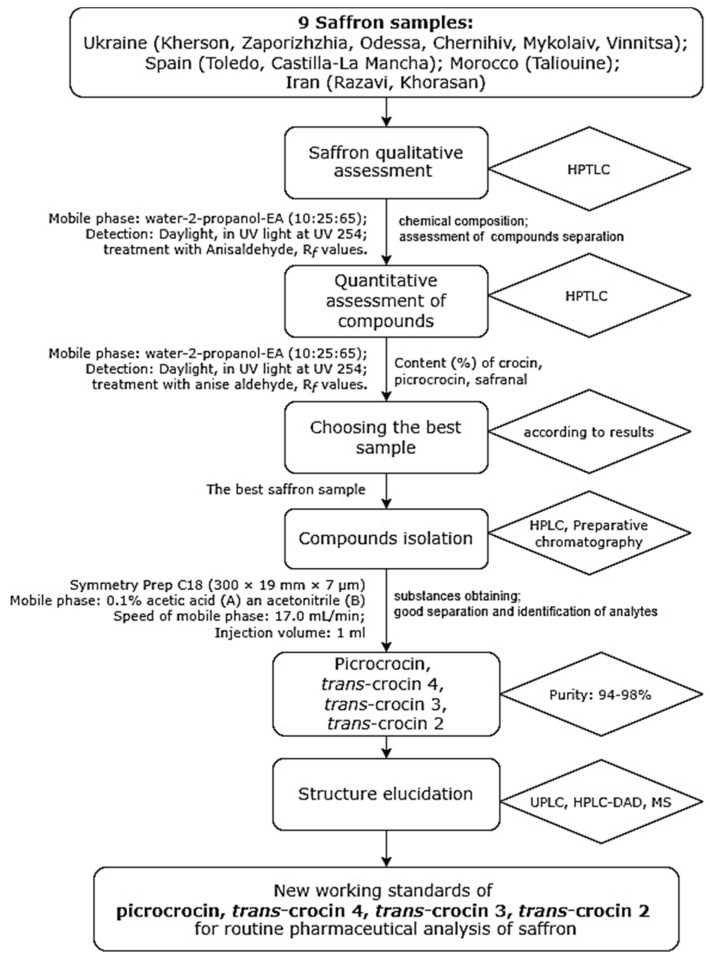
General view of the experiment design.

The reference standard of picrocrocin can be purchased, but the cost is very high. The individual standards of crocins are non-existent. There is the problem of the lack of reference standards or potential saffron pharmacological components. An important task for any pharmaceutical laboratory is to ensure that working standards are available for analysis, considering the latest advanced pharmacological developments based on stigma. This research aim was to propose the methodology for the isolation of picrocrocin and crocins (*trans*-4GG, *trans*-2G, *trans*-3Gg) as working standards for the routine pharmaceutical analysis of saffron. This aim included the following tasks: (1) assess the quantitative composition of saffron samples; (2) the simple, fast and precise quantification of crocins, safranal, and picrocrocin in *C. sativus* stigma by high-performance thin-layer chromatography (HPTLC); (3) the selection of a saffron sample with the maximum compounds content for further analysis; (4) the development of a preparative method for the isolation of crocins and picrocrocin; (5) the confirmation of compounds structure; (6) justifying the possibility of applying the compounds as working standards for the routine pharmaceutical analysis of saffron. The design of the experiment is shown in Figure 1.

## 2. Results

### 2.1. HPTLC Fingerprinting Profile

The accurate identification of herbal raw materials is very important for quality control and traceability followed by the determination of active components [31,32]. The use of saffron as a medicinal component obliges manufacturers to carry out quality control to exclude the possibility of adulteration or poor quality of the raw materials [33]. The modern analytical high-performance thin-layer chromatography (HPTLC) method has recently been increasingly used in the quality control analysis of herbal medicines with reproducible results [34,35]. Therefore, today the leading pharmacopoeias of the world in the monograph on medicinal plants are increasingly considering HPTLC methods as an alternative or replacement to thin-layer chromatography (TLC) methods. In this study, we analyzed the proposed TLC methods in the pharmacopoeias for the analysis of the authenticity of saffron and applied the HPTLC method for quality control.

All saffron samples have been pre-tested according to ISO 3632 and European Pharmacopoeia requirements to confirm their quality and safe use in further analysis. Quality control included determining the content of weight loss on drying, total ash, and the test of coloring power (the absorbance measured at 440 nm (crocin) is not less than 0.44. UV-vis spectrum analysis (Figure S1a), the solubility in distilled water, and determination of crocins, picrococins, and safranal content in samples’ ‘aqueous solution’ by UV-Vis spectrophotometry (Figure S1b). All samples responded to Ph. Eur. requirements and ISO Category I – II. All numerical values are presented in Table S1 of the Supplementary Materials. Further, the samples were used for HPTLC identification.

**Figure 2:**
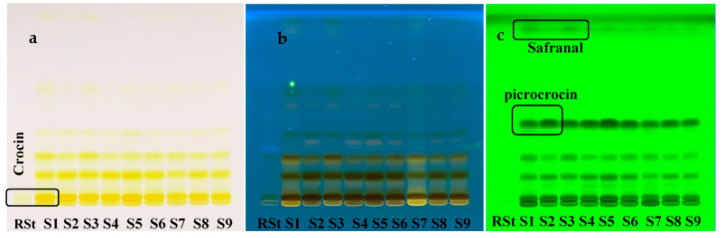
Chromatograms of saffron methanolic extracts in the HPTLC analysis: (**a**) under daylight and (**b**) at 366 nm before derivatization; (**c**) after derivatization with anisaldehyde at 254 nm.

The primary task of the study was to assess the composition and separation ability of saffron samples components by HPTLC. The requirements “Identification D. TLC identification” from the European Pharmacopoeia was chosen as the basis [21]. The methanolic extracts of saffron samples had good separation. The spot of the crocin reference standard (RSt) had a characteristic yellow fluorescence with R*_f_* 0.48 (Figure 2). The yellow zones of crocin and crocetin (R*_f_* 0.139–0.152) of samples S1-S9 in visible light are characteristic for *Crocus* stigma methanol extracts and both showed inhibition of fluorescence at UV-254 nm. The yellow-colored zone (R*_f_* 0.413–0.430) at daylight mode present in the saffron samples becomes dark violet-blue after derivatization with anisaldehyde, which confirmed the presence of picrocrocin in samples. Pale areas of safranal (4-hydroxycidocitral) may appear in the range R*_f_* of 0.661–0.683 after treatment of the chromatograms with anisaldehyde and heating at 100–105 °C for 5 min. The assignment of picrocrocin and crocetin spots without standards was carried out based on the R*_f_* values, exit times, order, and by literature data comparison [36,37]. Based on results obtained for Ukrainian samples (S1–S6), Spanish sample (S7), Moroccan sample (S8), and Iranian sample (S9) of saffron, it was proved that they have a similar chemical composition and good separating ability of the compounds under given conditions, but the compounds have different intensity or content compared to the main components. The picrocrocin reference standard (CAS No.138-55-6) is expensive and not every analytical laboratory can afford to purchase it for routine raw material authentication. Probably, therefore, in the Ph. Eur. 9.0 and in other pharmacopoeias of the world, there is no identification of this compound in saffron in comparison with the standard.

**Figure 3:**
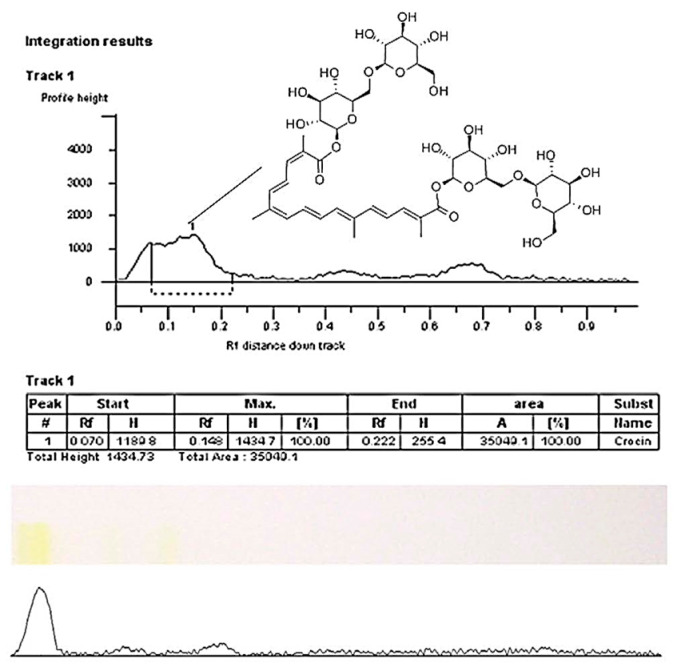
HPTLC chromatogram of crocin reference standard (5 mg/mL) and its chemical structure (peak 1).

**Figure 4:**
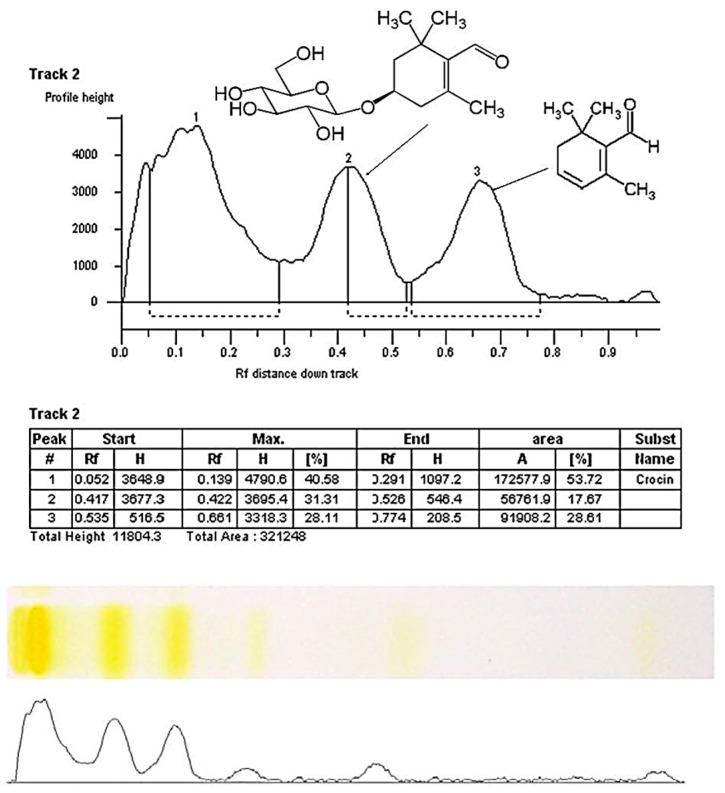
Typical HPTLC chromatogram of saffron sample (from Kherson region) at 366 nm before derivatization. Peak 1—crocin, peak 2—picrocrocin, peak 3—safranal.

The comparison of crocin reference standard on HPTLC chromatogram (Figure 3) and HPTLC profile a typical saffron stigma solution (Figure 4) shows that the selected peaks have a similar chemical nature, which confirms the presence of crocin in all saffron samples.

The advantage of the HPTLC method is the possibility of the quantitative evaluation of components content [30] and the possibility to perform screening of more samples in the same time. The calculation data of the main saffron metabolites content in analyzed samples are presented in Table 1. The results of the study showed that crocin content in Ukrainian saffron samples by the HPTLC method was 18–33%. For foreign saffron samples from Spain, Morocco, and Iran, content of crocin was 17%, 24%, and 32%, respectively. The picrocrocin content was the highest for samples from the Zaporizhzhia region, Ukraine, and from Iran, 24% and 26%, respectively. The content of safranal in Ukrainian samples was very different from 4 to 17%. The great difference in the amount of safranal, crocin, and picrocrocin in Ukrainian saffron samples is probably due to different environmental factors of the plant’s growing area [38] and the processing and drying of saffron [39]. According to the analysis, the saffron sample from Zaporizhzhia is the most optimal sample for developing SOP for obtaining working standards, as it has the highest content of crocins (33%), picrocrocin (24%), and safranal (13%) among the Ukrainian samples. A sample from Iran can also be considered for obtaining standards according to the high content of components, however, in the current work, we wanted to propose a method specifically for Ukrainian raw materials. For saffron from Ukraine, cultivation steps have been developed in accordance with the requirements of good agricultural and collection practices (GACP), which ensures the consistency of the composition of biologically active components [8].

The HPTLC method validation results are presented in Table 2. The Linear regression equation for crocin was 13,662x + 3425.2, with correlation coefficient *r*^2^ 0.9986 in the concentration range of 5000–39.06 μg/mL. Thus, the proposed HPTLC method can be successfully applied in pharmacopoeia analysis as a substitute for the TLC method for rapid screening of active constituents in *C. sativus* stigma and quality control. Further research includes obtaining the individual compounds from saffron stigma extract for use as working standards for analysis.

### 2.2. Preparative Isolation and Purification of Picrocrocin and Crocins

The profile of saffron chemical components has been thoroughly investigated by various modern methods in recent decades [10,11,15,18,39,40,41], but only a few studies [42,43,44,45,46,47] were devoted to the analysis of its individual components. 

Iborra et al. [42] made the first report on the separation of crocin, picrocrocin, and HTCC (2,6,6-trimethyl-4-hydroxy-1-carboxaldehyde-1-cyclohexene) by preparative TLC on aluminum oxide and the identification of compounds using spectroscopic methods.

Tarantilis et al. [43] used the HPLC-PDA for picrocrocin, crocins and safranal separation from saffron. D’Archivio et al. [41] used the UHPLC-DAD method for the separation and detection of crocins of *C. sativus* stigmas on the Kinetex C18 (Phenomenex) column. Sánchez et al. [44] suggested the solid-phase extraction method of the picrocrocin determining. This method included using the cyclohexane as a saffron extractant and the isolation of picrocrocin on the C_18_ column, and this was the first record of the compound isolation. Lautenschläger et al. [45] reported of the crocin-1 isolation from EtOH–water (2:8) extract using partition chromatography. Another study [46] described the extraction and purification of crocin with the crystallization method. Karkoula et al. [47] used the step gradient centrifugal partition chromatography method for crocetin, crocins, and picrocrocin isolation from the ethanolic saffron extract. However, the proposed method included multistage toxic reagent (petroleum ether, diethyl ether, methanol, water) extraction.

Corti et al. [48] also provided a method for the quantitative analysis and isolation of picrocrocin and crocetin from saffron. However, the authors use two different methods for each compound and use two columns for each isolation in further analysis. This method of isolation is difficult, time consuming, and costly. Karibi et al. [49] also proposed a method for picrocrocin and crocins isolation. Picrocrocin is obtained with a purity of only 91.13%. Both the first and the second authors use two different mobile phases for HPTLC analysis of the isolated compounds. By the comparison of current data research and previously published works [45,47], it can be seen that higher yield of compounds with higher purity (more than 97%). In our proposed method he lyophilization was used for better compound extraction. Lautenschläger et al. [45] focused on obtaining one of the components by the chemical elimination of glycoside (although this was a rather lengthy method).

However, there are no studies on the preparative isolation of picrocrocin and crocins after the lyophilization of saffron extract. To our knowledge, no one has purified these compounds and then performed an UPLC-ESI-MS/MS analysis of the components to establish their structure. The lyophilization process ensures rapid drying and, at the same time, comprehensive preservation of all biologically active compounds of the extract [50,51].

**Figure 5:**
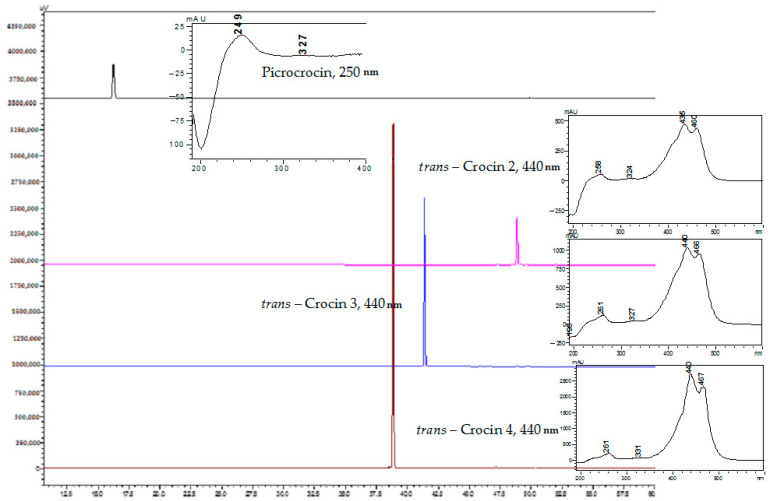
HPLC-DAD chromatograms of pure saffron compounds.

Saffron extract from Zaporizhzhia was lyophilized at a reduced temperature of –25 °C and under reduced pressure overnight and was then used for preparative isolation by high-performance liquid chromatography (HPLC). The method of the lyophilized drying of plant extracts ensures the minimum drying time and with that reserves sensitivity to heat compounds [51]. After lyophilization, four compounds were obtained using column Symmetry Prep C18 (300 × 19 mm × 7 µm). The approximate yield of each compound was about 2 mg. Picrocrocin and crocins (*trans*-4GG, *trans*-2G, *trans*-3Gg) were isolated with the purity of 97.75% and 94.12–98.41%, respectively. Figure 5 displays the UV-spectra and HPLC chromatograms of the detected compounds. The UPLC-ESI-MS/MS method was used for each compound to confirm the structure.

### 2.3. Structure Confirmation

**Figure 6:**
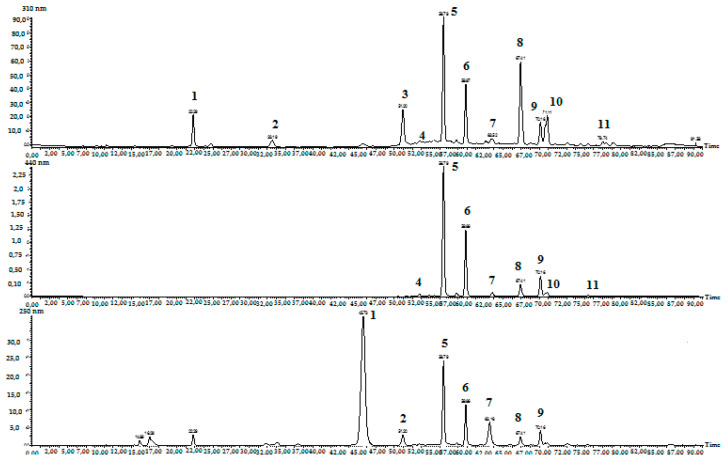
The typical HPLC chromatograms of methanol extract (50%) of saffron stigma recorded at 310 nm, 440 nm, 250 nm. The peak of compounds affiliation according to Tarantilis et al. (1995): **1**—picrocrocin; **2**—picrocrocin acid form; **3**—kaempferol diglycoside; **4**—*trans*-crocin-5; **5**—*trans*-crocin-4; **6**—*trans*-crocin-3; **7**—*cis*-crocin-5; **8**—*cis*-crocin-4; **9**—*trans*-crocin-2; **10**—*cis*-crocin-3; **11**—*cis*-crocin-2.

As results of the preparative chromatography method showed, several crocins and an important metabolite of *C. sativus* stigma picrocrocin were obtained. The chromatograms (Figure 6) of saffron methanol extract (50%) recorded at 310 nm, 440 nm, 250 nm showed the presence from 9 to 10 peaks. The peaks of compounds were identified and tentatively determined according to Tarantilis et al. [10] based on the time and sequence of output peaks, on UV spectra of *cis*-/*trans*-isomeric form of crocetin and *trans*-crocins. Saffron components present characteristic UV–Vis spectra, which are easily recorded during the HPLC separation of plant extract, online with the aid of a diode-array detector. Most compounds with retention times in the range of 50–80 min exhibit a typical UV–visible spectrum of crocins and a strong absorption band at 440 nm characteristic for crocetin esters. In the methanol extract of saffron sample from Ukraine, the following components were predetermined: *trans*-crocins (t-5tG, t-4GG, t-3Gg, t-2G) and *cis*-crocins (c-5tG, c-4GG, c-3Gg, c-2G). The UV spectrum of picrocrocin has a characteristic broad absorption band at 250 nm due to the presence of *a*, *β*-unsaturated cycloaldehyde in its structure [10]. Flavonoids were identified in saffron stigma previously, and these were mainly glycosides and diglycosides of kaempferol and quercetin [52]. Kaempferol diglycoside was identified in the current saffron extract on the basis of UV data. As the result, a comparison of the UV–visible spectra of crocins reveals their *trans*- and 13-*cis*-isomers. The 13-*cis* isomer of crocins has an additional band at approximately 350 nm, which is the exclusive band for the 13-*cis* isomers. In this case, crocin-3 and crocin-2 are *trans*-isomers, since there is no additional band. In Ukrainian saffron samples, *cis*-crocin 1 (c-1g) and *trans*-crocin 2′ (t-2gg) were not detected, that can be used for the geographical traceability of plant raw material [41]. Thus, as the result of separation, the picrocrocin and crocins (*trans*-4GG, *trans*-2G, *trans*-3Gg) were obtained from Ukrainian saffron for the first time. Those compounds have high purity and can be used as working standards for routine analytical work. The application of the proposed preparative HPLC method for the crocins and picrocrocin isolation from *Crocus* stigma will probably be useful for the pharmaceutical industry.

For structure elucidation of isolated compounds (picrocrocin, crocin-4, crocin-3, and crocin-2) from saffron methanolic extracts, the UPLC-ESI-MS/MS method was used. The use of electrospray mass spectrometric (ESI-MS) detection in the negative ion mode makes it possible to obtain mass fragmentation of each compound as an additional method for confirming their structure [53]. Their retention times (tR), molecular weights, and MS/MS data are shown in Table 3, respectively. In the ESI–MS spectrum of picrocrocin (C_16_H_26_O_7_, *m*/*z* 330) showed two adduct ions (with formic acid) at *m*/*z* 375 [M − H + HFA]^−^ and 365.2 [M − H + HCl]^−^ (Figures S2 and S3 of the Supplementary Material). The absorption maximum of picrocrocin was observed at 249 nm.

The major component of saffron was crocin-4, crocetin esterified with one gentiobiose at each end, the compound (calculated for C_44_H_64_O_24_, *m*/*z* 976) produced the [M − H]^−^ ion at *m*/*z* 975 at 5.64 min and exhibited absorption at maxima 261, 331, 440, and 467 nm. During the formation of adducts with formic acid in the mass spectrum of crocin 4, the peaks formed with *m*/*z* 1021.0 were identified as [M − H + HA]^−^, 978.3 [M − H + HCl]^−^, and 697.9 [M – H − Gnt + HA]^−^. The presented identification of mass ions is also characteristic of compounds containing one chlorine atom and is consistent with previously published observations of Lech et al. [53].

Crocin-3 (C_38_H_54_O_19_, *m*/*z* 814), crocetin esterified with one gentiobiose at one end and one glucose at the other, *trans-* and *cis-* isomers showed the [M − H]^−^ ion at *m*/*z* 813. The UV-spectrum registered for crocin-3 exhibits absorption maxima at 261, 327, 440, and 466 nm.

The use of formic acid as a mobile phase modifier gave an intense signal at *m*/*z* 859.2 [M − H + HA]^−^, and also showed the presence of adduct ions [M − H + HA + NFaA]^−^ at *m*/*z* 927.7 and [M − H + HCl]^−^ at *m*/*z* 849.5. Other ions [M – H − Glc]^−^ at *m*/*z* 651.2 were assigned to the glucoside residue and [M – H − Gnt]^−^ at *m*/*z* 489.2 to gentiobiosyl, respectively.

Crocin-2 (C_33_H_44_O_14_, *m*/*z* 652) crocetin esterified with one gentiobiose at one end and a free carboxylic group at the other showed the [M – H]^–^ ion at *m*/*z* 651 at 7.29 min. The compound has similar ions fragmentation as a crocin-4 and crocin-3, and also has four maxima absorption at 258, 324, 435, and 460 nm characteristic of crocins-family. The obtained data were in a good agreement with previously published research [53]. The presented data provide reliable confirmation of the structure of the obtained crocins and picrocrocin based on the analysis of mass spectra.

## 3. Materials and Methods

### 3.1. Plant Material and Reagents

*C. sativus* stigma samples were harvested in 2019 in different Ukraine regions: Kherson (S1), Zaporizhzhia (S2), Odesa (S3), Chernihiv (S4), Mykolaiv (S5), and Vinnitsa (S6). The commercial saffron samples were purchased from Spain (Camuñas, Toledo, Castilla-La Mancha, “Spanish Saffron”) (S7), Morocco (Taliouine region) (S8), and Iran (North Khorasan province, Razavi, ATR Afarin Saffron) (S9). Samples were identified by Dr. Mykhailenko (voucher specimens No. 20191–20198). All the numerical indicators of saffron samples quality were verified according to ISO/TS 3632 standards (ISO/TS 3632, 2010/2011). For analysis were used: HPLC grade methanol, acetonitrile, and acetic acid (purity ≥ 98.0%) (Sigma-Aldrich GmbH, Switzerland), and crocin reference standard with a purity ˃ 90% (CAS number 42553-65-1, Sigma-Aldrich, St. Louis, MO, USA).

### 3.2. Sample Preparation

Identification test. Saffron samples were ground using liquid nitrogen. Each powdered sample weighed 100 mg, and 50% methanol (10 mL) was used for extraction. The flask was placed in an ultrasonic bath (Wise Clean WUC-A06H, Witeg Labortechnik GmbH, Wertheim, Germany) at room temperature (20 ± 2 °C) for 15 min. After extraction, extracts were centrifuged and the supernatant was used for chromatography (HPTLC).

Isolation method. Saffron sample from Zaporizhzhia (Ukraine) was used for further analysis. The extract (100 mL; 50% methanol) of the chosen saffron sample was obtained similarly to the description above. Then, it was lyophilized at a reduced temperature of –25 °C and under reduced pressure overnight (Liofilizator, Telstar lyoquest, HT 40, Lisboa, Portugal). The obtained lyophilized extract was used for preparative isolation of picrocrocin and crocin derivatives by HPLC and UPLC.

### 3.3. HPTLC Conditions

For analysis, we used Camag TLC system: win CATS 1.2.2 software, Camag^®^ Linomat 5 autosampler, TLC scanner 3, Camag^®^ TLC Visualizer. Separation was performed on TLC precoated silica gel 60F_254_ plates 10 × 10 cm (Merck 157 CO., Darmstadt, Germany). As a mobile phase was applied ethyl acetate: isopropanol: water (10:25:65 by *v*/*v*). Application conditions: the width—5 mm (5 μL), rate—50 nL/s. TLC plates were dried on the open air, then heated up on Camag HPTLC plate heater III at 60 °C for about 5 min. The scanning was performed at daylight, at 366 nm, at 254 nm (without treatment), as well as at 254 nm after plate derivatization with a spray reagent (anisaldehyde solution) and heating at 100–105 °C for 5–10 min. Quantitative analysis of the compounds was done by scanning the plates. The amount (%) of picrocrocin and safranal are presented in terms of crocin standard.

### 3.4. Compounds Isolation by HPLC

The Waters preparative HPLC Purification System (Milford, MA, USA) was used for preparative chromatography. The system comprised a 2767 Sample Manager, binary 1525 HPLC pumps, a 2488 UV detector and was controlled by Mass Lynx software version 4.1. The column isolation was a Symmetry Prep C18 (300 × 19 mm × 7 µm). HPLC conditions: mobile phase speed—17.0 mL/min; injection volume—1 mL; column temperature—20 °C; the mobile phase—a mixture of 0.1% acetic acid (A) and acetonitrile (B) with the linear gradient program 0–10 min, 5–15% B; 10–36 min, 15–20% B; 36–58 min, 20–40% B; 58–70 min, 40–50% B; 70–78 min, 50% B; 78–80 min, 50–95% B. The retention time of picrocrocin in these conditions was 45.73 min, for crocin—57 min, crocin (2)—60.4 min, crocin (3)—70.65 min. UPLC, HPLC, UV-vis, and MS studies were used for structural confirmation of picrocrocin and crocins (*trans*-4GG, *trans*-2G, *trans*-3Gg).

### 3.5. UPLC-ESI-MS/MS Conditions

Components separation has been performed by UPLC-ESI-MS/MS analysis [54] using the ACQUITY UPLC H-Class streamlined system (Waters, Milford, MA, USA) with BEH C18 (50 × 2.1, particle size 1.7 µm) (Merck Millipore, Darmstadt, Germany). The mobile phase consisted of 0.1% formic acid water solution (A) and acetonitrile (B). The gradient elution was as follows: initial—5%, 3 min—30%/B, 7 min—50%/B, 7 to 8 min—95%/B, 15 to 16 min—5%/B. The flow rate was 0.5 mL/min. The mass spectrometric detection was performed [55] in the positive ion mode (1.5 kV capillary voltage), collision energy varied in range from 6eV to 20 eV, and cone voltage was selected from 8 V to 38 V. The following parameters of electrospray ionization were applied: source temperature was 150 °C, desolvation temperature was 350 °C, desolvation gas flow was 650 L/h, and cone gas flow was 25 L/h. To obtain MS/MS data, we used Xevo TQD triple quadrupole mass spectrometer detector (Waters Millford, MA, USA).

### 3.6. HPTLC Method Validation

Method validation was performed based on the International Conference on Harmonization [56] Q2B guidelines and the following parameters were taken into account: linearity, the limit of detection (LOD), and limit of quantification (LOQ) for crocin standard of the method (Table 2).

### 3.7. Statistical Analysis

All experiments were performed in triplicate and the results were expressed as mean ± standard deviation (SD) for three replications of each sample. Statistical analysis of HPTLC analysis was performed by one-way analysis of variance (ANOVA) followed by Tukey’s multiple comparison test with the software package Prism v.5.04 (GraphPad Software Inc., La Jolla, CA, USA). The value of *p* < 0.05 was taken as a level of significance.

## 4. Conclusions

This study suggests the simple, fast, selective, and environmentally friendly preparative chromatography method of picrocrocin and crocins (*trans*-4GG, *trans*-2G, *trans*-3Gg) is isolation and purification from saffron. The resulting compounds may be used as working standards for routine quality control. In addition, the proposed HPTLC method showed good separation of 11 tentative detected saffron compounds. A comparative analysis by the HPTLC method of nine different saffron samples showed a similar chemical composition and good quality of the samples. Moreover, the proposed method was used to determine the amount of main compounds (safranal, crocin, picrocrocin) in *Crocus* stigma. Reference standard crocin showed good linearity of the calibration curves, low detection limit, and quantification limit. The obtained data show that the presented HPTLC method is simple, precise, accurate, and will be quite suitable for regular quality control of saffron in pharmaceutical analysis.

## Figures and Tables

**Table 1:** The amount of main compounds (%) in the methanolic saffron extracts.

Track *	Standart/Saffron Origin	Crocin		Picrocrocin		Safranal	
		R*_f_*	Content, %	R*_f_*	Content, %	R*_f_*	Content, %
RSt	Crocin standard	0.148	-	-	-	-	-
S1	Kherson, Ukraine	0.139	29.12 ± 3.90	0.422	9.92 ± 2.50	0.661	15.51 ± 4.69
S2	Zaporizhzhia, Ukraine	0.139	33.25 ± 4.39	0.413	24.20 ± 4.63	0.665	12.89 ± 2.39
S3	Odessa, Ukraine	0.143	30.00 ± 3.99	0.426	21.67 ± 3.21	0.674	17.14 ± 2.17
S4	Chernihiv, Ukraine	0.143	17.80 ± 2.42	0.426	21.02 ± 3.08	0.678	4.10 ± 0.97
S5	Mykolaiv, Ukraine	0.139	27.37 ± 2.86	0.413	20.49 ± 2.56	0.674	4.13 ± 1.30
S6	Vinnitsa, Ukraine	0.148	26.08 ± 2.65	0.422	19.58 ± 2.83	0.678	4.81 ± 1.49
S7	Toledo, Spain	0.126	17.26 ± 3.37	0.430	13.49 ± 2.77	0.674	10.17 ± 2.08
S8	Taliouine, Morocco	0.152	24.47 ± 3.54	0.430	19.92 ± 2.75	0.678	5.08 ± 1.53
S9	Razavi, Iran	0.152	32.34 ± 4.61	0.430	26.55 ± 3.59	0.683	7.16 ± 1.69

* The names correspond to the order of the tracks on the chromatograms in Figure 2.

**Table 2:** The data of calibration curve, LOD, and LOQ of crocin of the proposed HPTLC method.

Compound	Calibration Curve ^a^	Correlation Coefficient (*r*^2^)	Concentration Range, (μg/mL)	LOD ^b^, (ng/mL)	LOQ ^c^, (ng/mL)
Crocin	y = 13,662x + 3425.2	0.9986	5000–39.06	0.22	0.66

^a^ compound concentration (mg/mL); y, peak area; the ^b^ LOD and ^c^ LOQ under the present chromatographic conditions were determined at S/N of 3 and 10, respectively.

**Table 3:** Chromatographic and spectral data of pure components from saffron.

Compound	Abbre-viation *	HPLC, R_time_, min	UV Max, nm	UPLC-MS, R_time_, min	[M − H]^−^, *m*/*z*	Fragment Ions, *m*/*z*	Adduct Ions (Formic Acid) [M − H + HAA]^−^	Molecular Mass, g/moL	Purity, % **
Picrocrocin	-	45.70	249	4.42	N/O	N/O	375.1 [M − H + HA]^−^ 365.2 [M − H + HCl]^−^	330	97.75
Crocin 4 (*trans*-crocetin di( *β*-D-gentiobiosyl) ester)	t-4GG	57.00	261, 331, 440, 467	5.64	975.2	651.1 [M – H − Gnt]^−^	1021.0 [M − H + HA]^−^ 978.3 [M − H + HCl]^−^ 697.9 [M − H − Gnt + HA]^−^	976	98.41
Crocin 3 (*trans*-crocetin (*β* -D-glucosyl)-( *β*-D-gentiobiosyl) ester)	t-3Gg	60.42	261, 327, 440, 466	6.35	813.1	651.3 [M − H − Glc]^−^ 489.3 [M − H − Gnt]^−^	927.7 [M − H + HA + NaA]^−^ 859.2 [M − H + HA]^−^ 849.5 [M − H + HCl]^−^	814	97.85
Crocin 2 (*trans*-crocetin(*β* -D-gentibiosyl) ester)	t-2G	70.56	258, 324, 435, 460	7.29	651.4	N/O	697.0 [M − H + NaA]^−^ 812.1 [M − H + HA]^−^	652	94.12

* Abbreviation of known crocins were presented according to Sánchez et al. 2009 [44]. ** Purity, percentage of the total peak area in the chromatogram. N/O—not observed.

## Data Availability

Not applicable.

## Supplementary Materials

The following supporting information can be downloaded at: https://www.mdpi.com/article/10.3390/molecules27134286/s1, Table S1 and Figure S1 “Quality characteristics of saffron samples”. Figure S2 “Structural formulas of isolated compounds”, Figure S3–S10: UPLC-MS/MS chromatograms and MS data of working standards (picrocrocin, *trans*-crocin 4, *trans*-crocin 3G, *trans*-crocin 2).

## Sample Availability

Not available.
