# Supplementary material for: Effective Isolation of Picrocrocin and Crocins from Saffron: From HPTLC to Working Standard Obtaining
Source: Molecules. 2022 Jul 3;27(13):4286. doi: 10.3390/molecules27134286 (PMC9267943; doi:10.3390/molecules27134286)
Supplement: Supplementary file 1 [file molecules-27-04286-s001.zip › molecules-1763485-supplementary.pdf]

molecules

Supplementary material

Effective isolation of picrocrocin and crocins from saffron: from HPTLC to working standard obtaining

Laurynas Jarukas [1], Konradas Vitkevicius [1], Olha Mykhailenko [2], Ivan Bezruk [2], Victoriya Georgiyants [2] and Liudas Ivanauskas [1*]

[1] Department of Analytical and Toxicological Chemistry, Lithuanian University of Health Sciences, A. Mickeviciaus str. 9, LT-44307 Kaunas, Lithuania; laurynas.jarukas@lsmuni.lt (L.J.); Konradas.Vitkevicius@lsmuni.lt (K.V.); liudas.ivanauskas@lsmuni.lt (L.I.)
[2] Department of Pharmaceutical Chemistry, National University of Pharmacy, Valentynivska, str. 4, 461168 Kharkiv, Ukraine, mykhailenko.farm@gmail.com (O.M.), vania.bezruk@gmail.com (I.B.), vgeor@nuph.edu.ua (V.G.)
* Correspondence: mykhailenko.farm@gmail.com; Tel.: (+380509277385) Dr. Olha Mykhailenko

Table of Contents

Table S1. Quality characteristics of saffron samples according to ISO 3632 and Ph. Eur. 9th Edition……………..2

Figure S1. Typical UV absorption spectra of the tested solutions of *Crocus sativus* stigma (Kherson regain): A. obtained under the conditions of the ISO 3632 method; B. obtained under the conditions of the method Ph. Eur. 9th Edition. 2

Figure S2. Structural formulas of isolated and identified picrocrocin and *trans*-crocetin esters and substituents at R1 and R2 2

Figure S3. UPLC-MS/MS chromatogram of pure picrocrocin with retention time 4.41 from the methanol extract of saffron stigma 3

Figure S4. Mass spectrum of picrocrocin from the methanol extract of saffron stigma 4

Figure S5. UPLC-MS/MS chromatogram of pure Crocin-4 (*trans*-crocin 4) with retention time 5.64 from the methanol extract of saffron stigma. 5

Figure S6. Mass spectrum of trans-crocin 4 from the methanol extract of saffron stigma. 6

Figure S7. UPLC-MS/MS chromatogram of pure Crocin-3 (*trans*-crocin 3) with retention time 6.34 of the methanol extract of saffron stigma..7

Figure S8. Mass spectrum of trans-crocin 3 from the methanol extract of saffron stigma a. 8

Figure S9. UPLC-MS/MS chromatogram of pure *trans*-crocin 2 with retention time 7.29 from the methanol extract of saffron stigma. 9

Figure S10. Mass spectrum of *trans*-crocin 2 from the methanol extract of saffron stigma..10

Table S1. Quality characteristics of saffron samples according to ISO 3632 and Ph. Eur. 9th Edition

Saffron Sample	Moisture and volatile matter content, %	Total ash, %	Colouring power ac. Ph.Eur.[a], A	Picrocrocin, 257 nm	Safranal, 330 nm	Crocin, 440 nm	Soluble extract in cold water, %	Saffron Category [b]
Kherson, Ukraine	5.80 ± 0.12	3.84 ± 0.01	0,566	85.10 ± 1.28	38.10 ± 0.39	235.22 ± 4.14	60	I
Zaporizhia, Ukraine	9.04 ± 0.15	6.41 ±0.03	0,575	96.14 ± 1.10	32.16 ± 0.02	262.26 ± 4.05	58	I
Odesa, Ukraine	6.71 ± 0.65	6.00 ± 0.05	0,446	96.02 ± 1.12	35.15 ± 0.03	243.22 ± 1.02	55	I
Chernihiv, Ukraine	7.03 ± 0.08	4.50 ± 0.03	0,485	101.05 ± 1.15	34.10 ± 0.15	258.03 ± 4.15	58	I
Mykolaiv, Ukraine	11.67±0.26	8.45 ± 0.20	0,460	90.60 ± 1.60	21.24 ± 0.21	185.16 ± 3.25	58	II
Vinnitsa, Ukraine	7.22 ± 0.14	4.81 ± 0.08	0,550	62.30 ± 1.10	39.36 ± 0.55	139.75 ± 2.47	60	II
Castilla-La Mancha, Spain	8.01 ± 0.18	6.34 ± 1.32	0,560	78.22 ± 1.32	56.25 ± 1.03	133.30 ± 2.17	60	II
Taiyuan, Morocco	2.33 ± 0.01	1.05 ± 0.07	0,440	94.15 ± 1.17	37.20 ± 0.54	209.35 ± 3.52	56	I
Khorasan, Iran	6.52 ± 0.28	4.15 ± 1.30	0,520	77.16 ± 1.30	58.02 ± 1.05	214.05 ± 3.84	60	I

[a]The absorbance measured at 440 nm (crocin) is not less than 0.44. UV-vis spectrum analysis; [b]Categories according to the ISO 3632 (saffron filaments): Category I: picrocrocin > 70, safranal 20-50, crocins > 200; II: picrocrocin >55, safranal 20-50, crocins >170; III: picrocrocin >40, safranal 20-50, crocins >120.

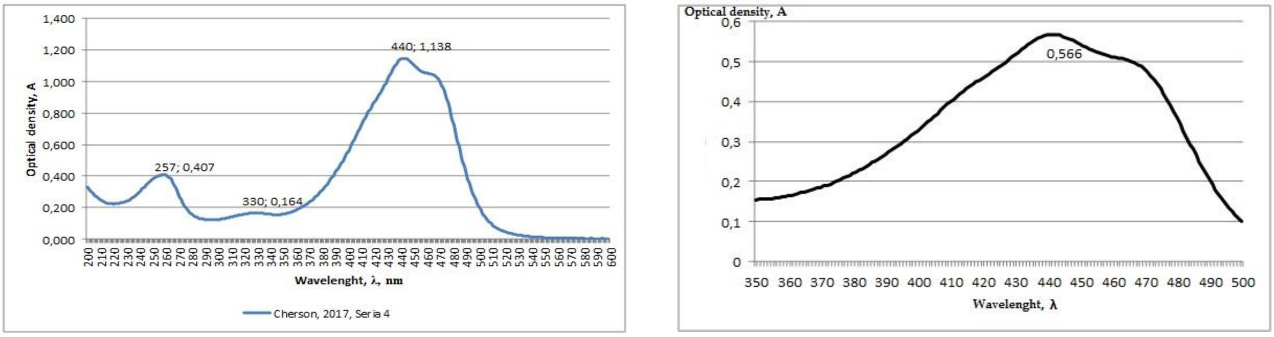

A B

Figure S1. Typical UV absorption spectra of the tested solutions of *Crocus sativus* stigma (Kherson regain): A. obtained under the conditions of the ISO 3632 method; B. obtained under the conditions of the method Ph. Eur. 9.0. Spectrophotometer 60S UV-Visible, Thermo Scientific (USA).

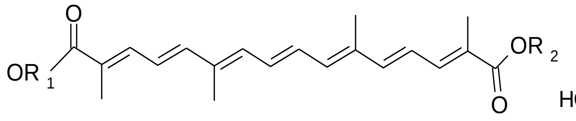

Moiety distridution (R_1/R_2)	Isomer	Crocetin esters*
gentiobioside/gentiobioside	*trans*	*trans*-4-GG
gentiobioside/glucoside	*trans*	*trans*-3-Gg
gentiobiose/hydrogen	*trans*	*trans*-2-G

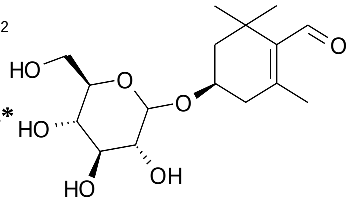

Picrocrocin

Figure S2. Structural formulas of isolated and identified picrocrocin and *trans*-crocetin esters and substituents at R_1 and R_2. Nomenclature according to Carmona et al. (2006).

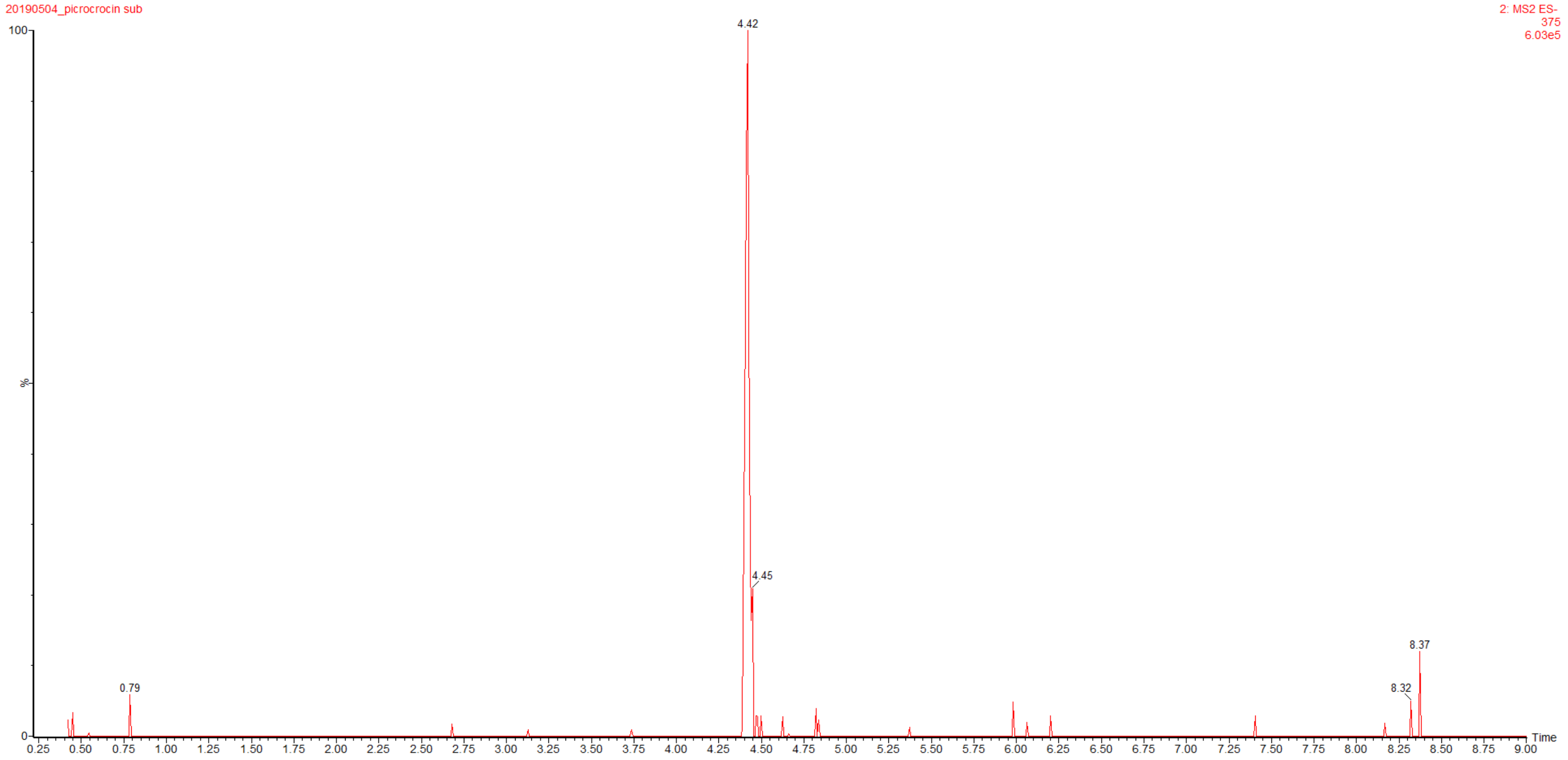

Figure S3. The UPLC-MS/MS chromatogram of pure picrocrocin with retention time 4.41 from the methanol extract of saffron stigma.

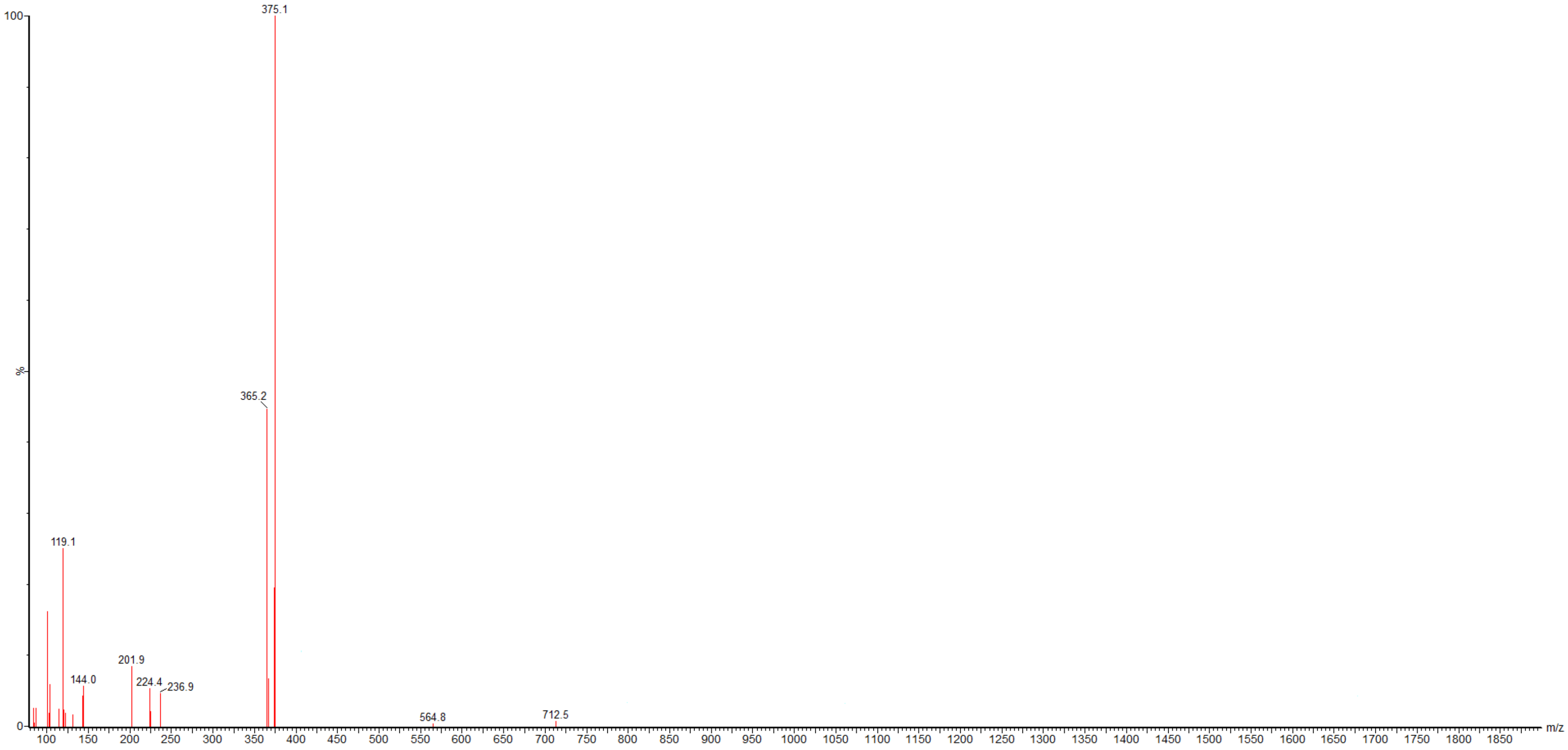

Figure S4. Mass spectrum of picrocrocin from the methanol extract of saffron stigma.

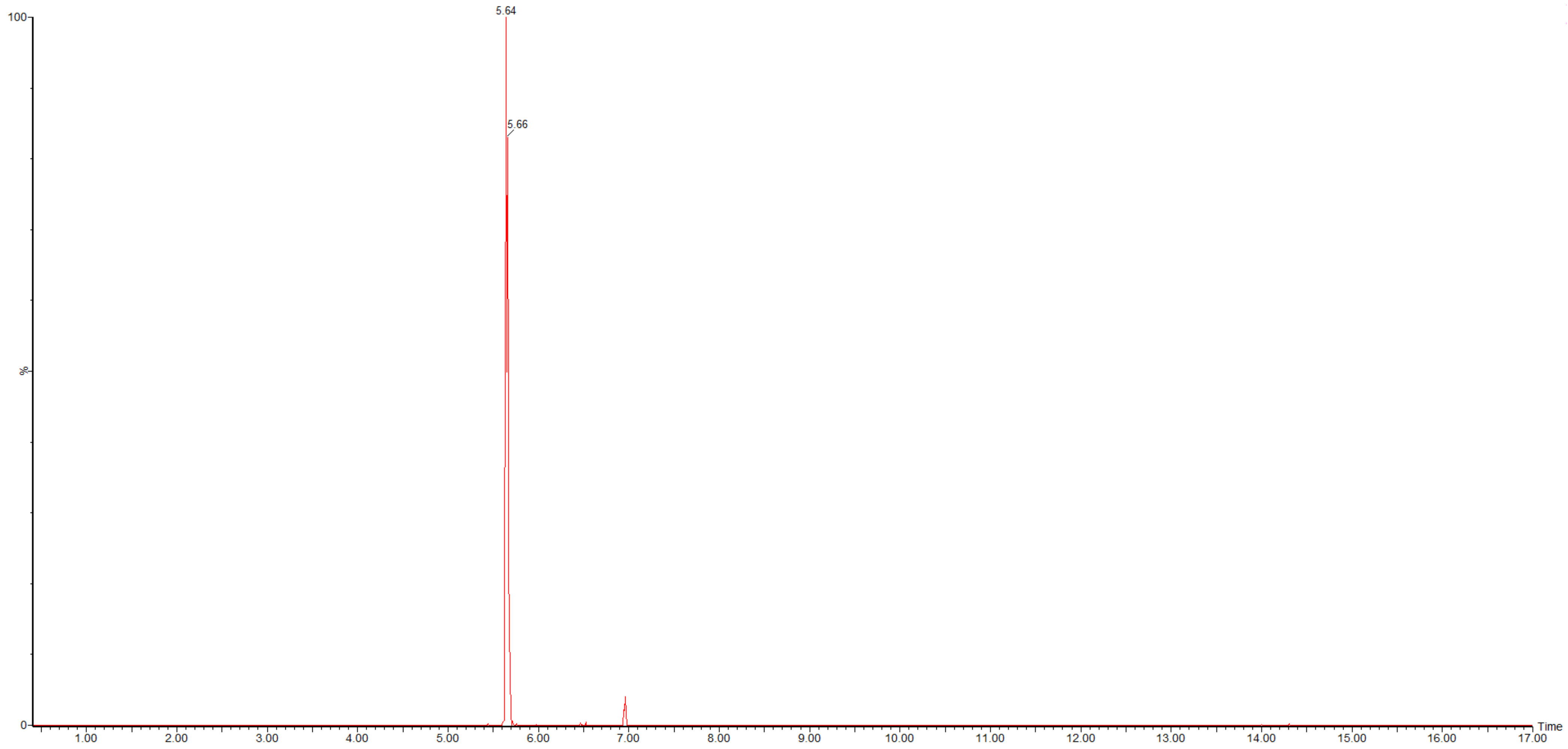

Figure S5. The UPLC-MS/MS chromatogram of pure Crocin-4 (*trans*-crocin 4) with retention time 5.64 from the methanol extract of saffron stigma.

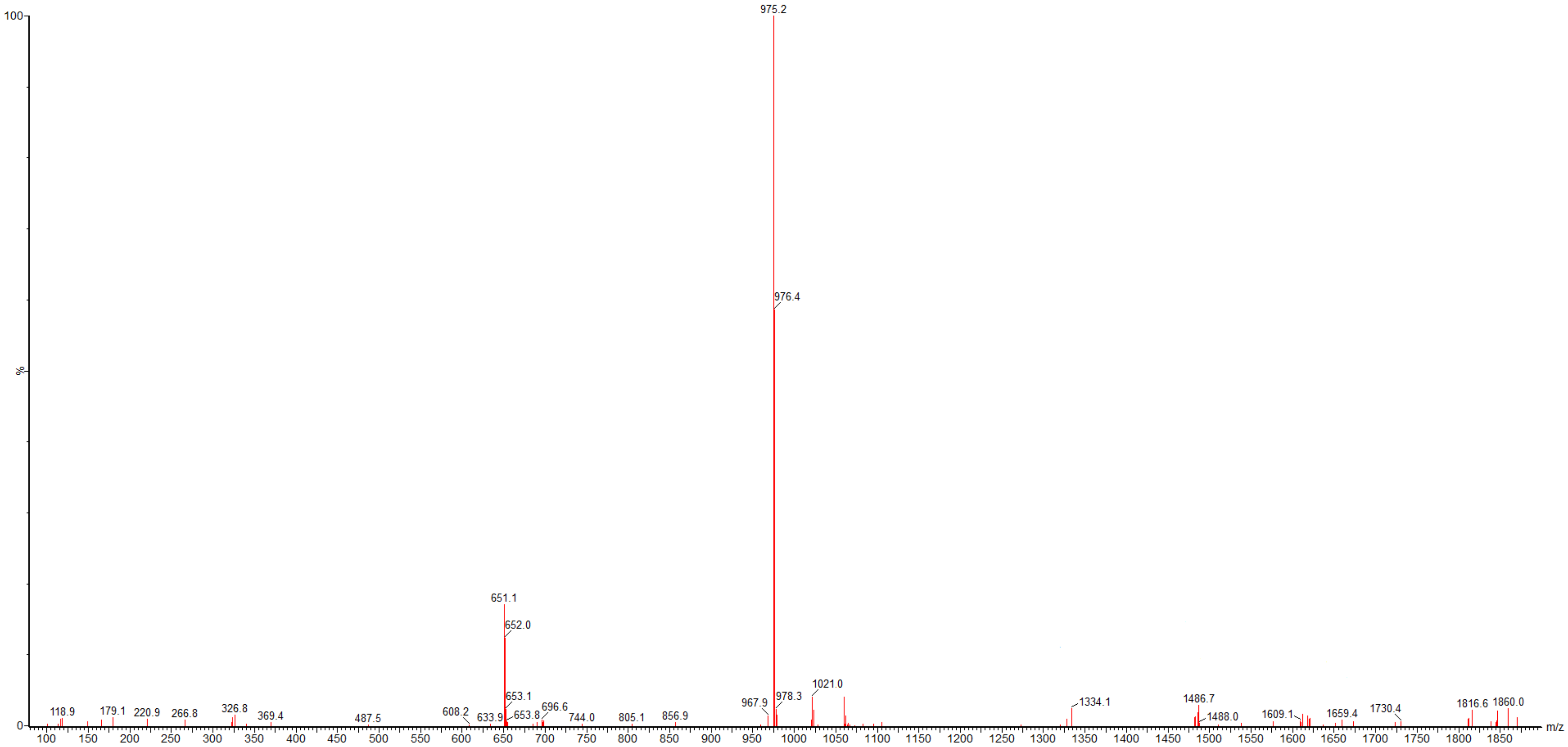

Figure S6. Mass spectrum of *trans*-crocin 4 from the methanol extract of saffron stigma.

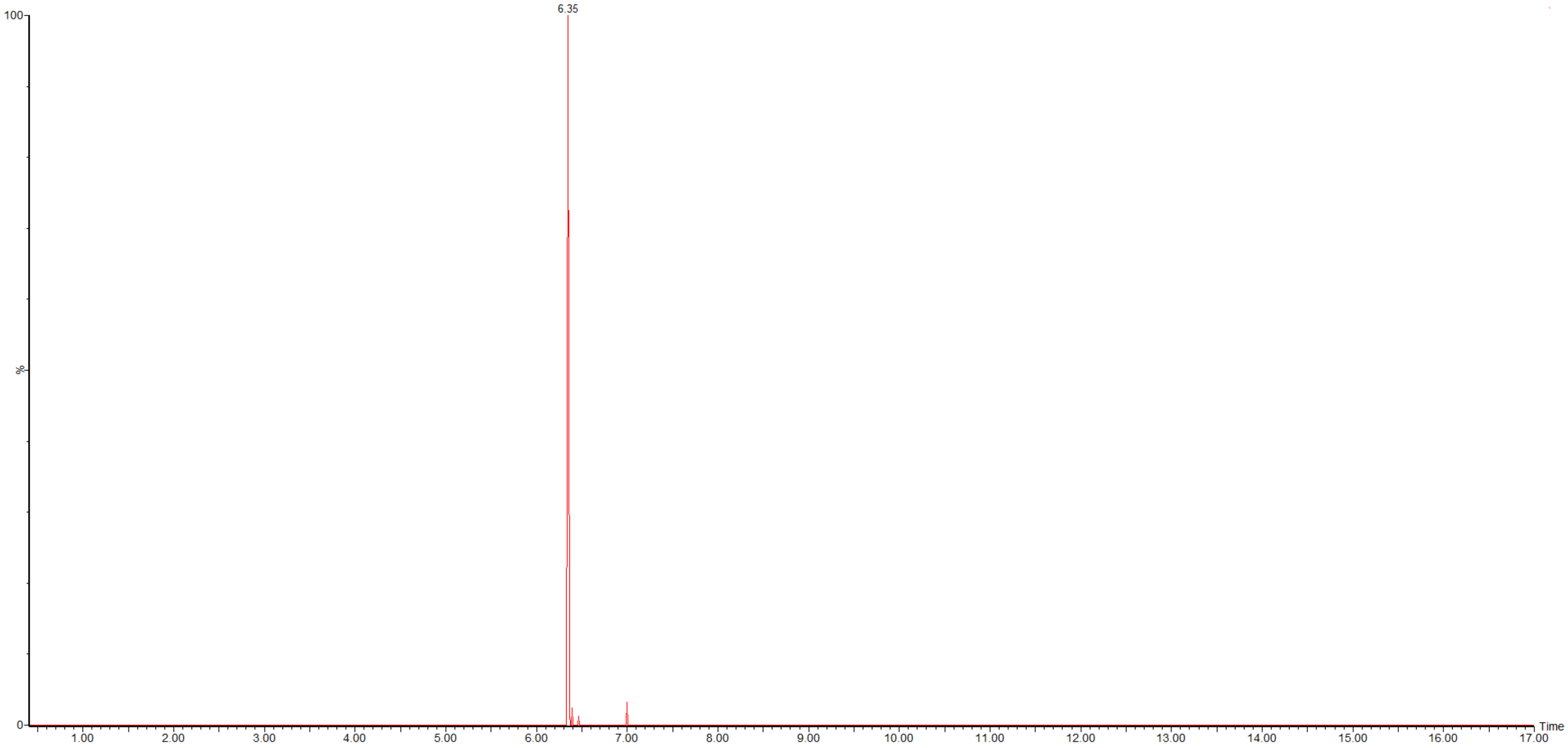

Figure S7. The UPLC-MS/MS chromatogram of pure Crocin-3 (*trans*-crocin 3) with retention time 6.34 of the methanol extract of saffron stigma.

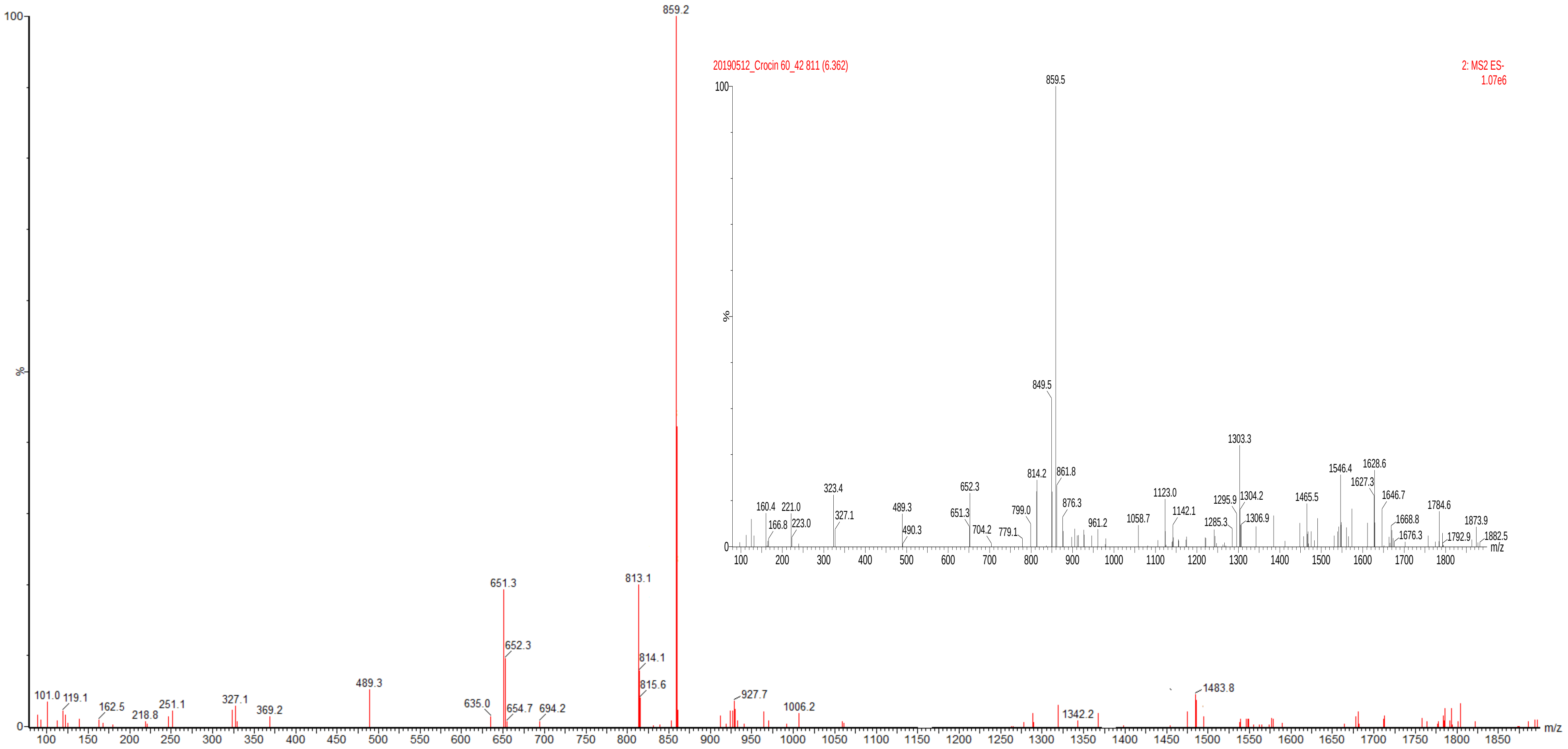

Figure S8. Mass spectrum of *trans*-crocin 3 from the methanol extract of saffron stigma.

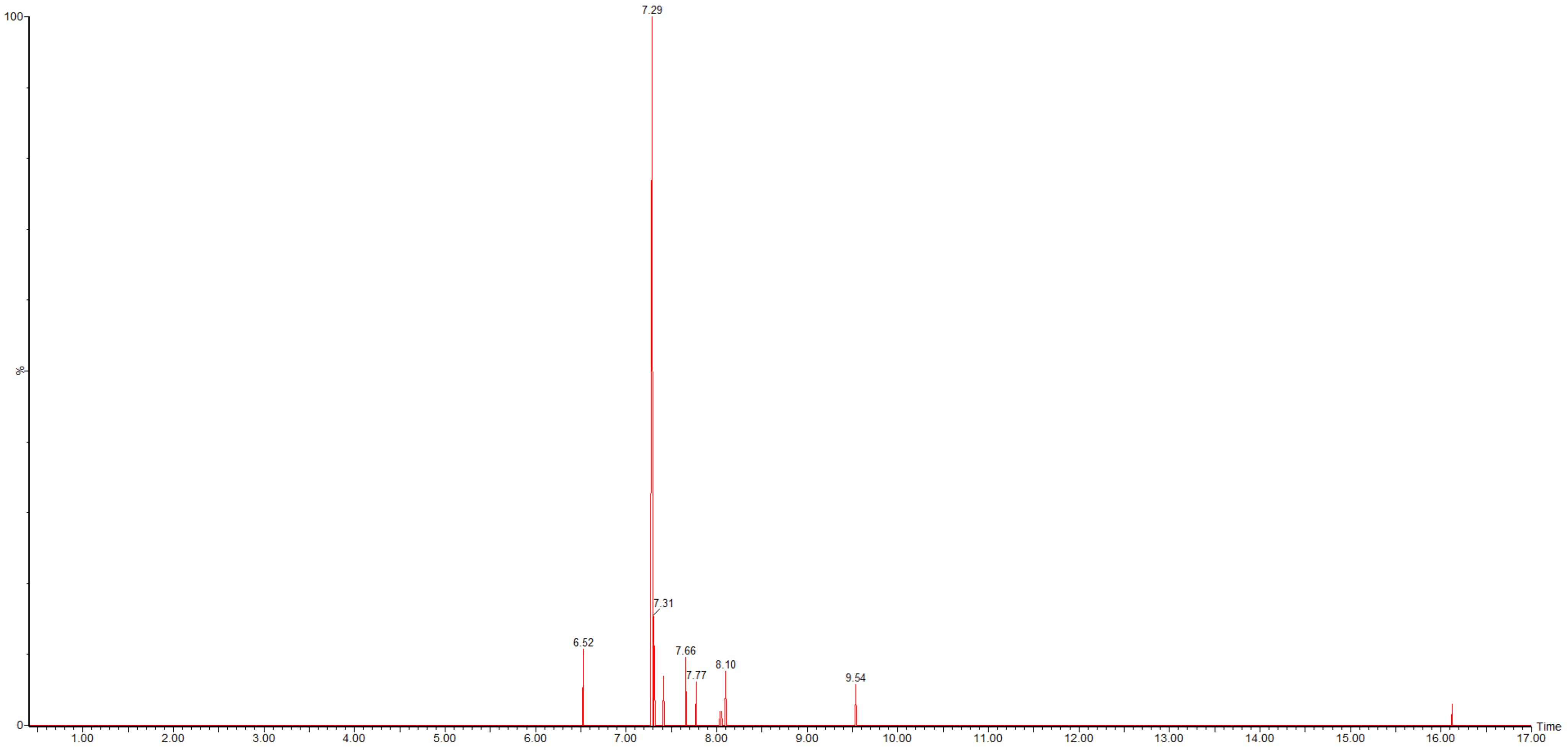

Figure S9. The UPLC-MS/MS chromatogram of pure *trans*-crocin 2 with retention time 7.29 from the methanol extract of saffron stigma

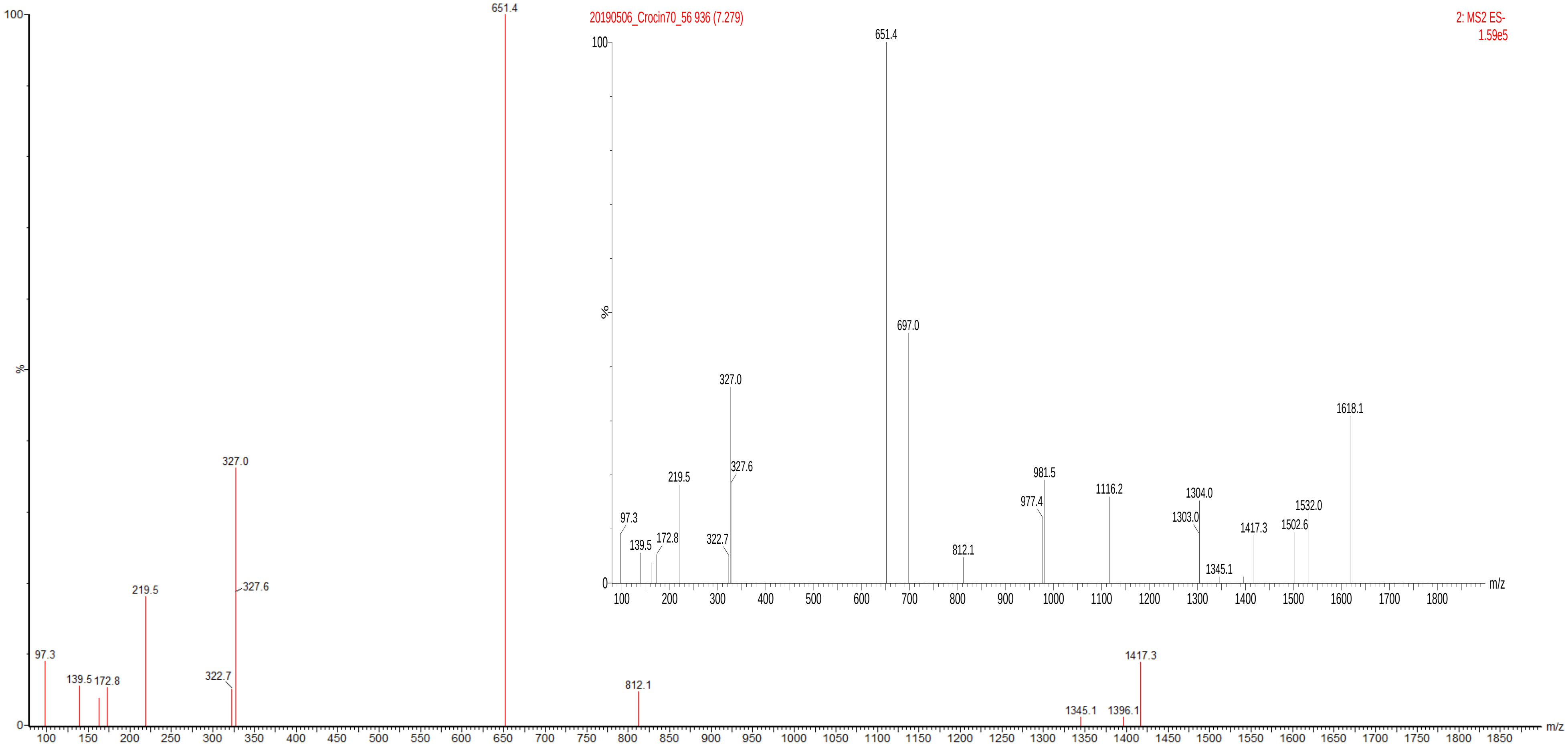

Figure S10. Mass spectrum of *trans*-crocin 2 from the methanol extract of saffron stigma.
